# Case series: Continued remission of PTSD symptoms after discontinuation of prazosin

**DOI:** 10.3389/fpsyt.2023.1172754

**Published:** 2023-04-21

**Authors:** Christie Richardson, Jonathan Yuh, Jing Su, Martin Forsberg

**Affiliations:** Department of Psychiatry, Rowan-Virtua School of Osteopathic Medicine, Mount Laurel, NJ, United States

**Keywords:** post-traumatic stress disorder, prazosin, trauma, nightmares, flashbacks, psychopharmacology

## Abstract

Post-traumatic stress disorder is a debilitating chronic illness that affects 6 out of 100 adults after a severe trauma. The alpha-adrenergic antagonist prazosin, which is prescribed off-label for flashbacks and nightmares due to trauma, is often continued indefinitely due to reports of symptoms returning upon discontinuation. There is no standard guidance for a trial of discontinuation of prazosin due to intolerance or side effects. In this case series, three patients are started on prazosin leading to remission of trauma-related symptoms, and symptoms continue to remit after treatment for an average of about 2 years followed by discontinuation of the medication. There are many similarities in these case reports which serve to provide guidance as to when a trial of prazosin discontinuation may be warranted.

## Introduction

Post-traumatic stress disorder (PTSD) is a chronic illness defined as more than 1 month of intrusion symptoms, avoidance, negative alterations in cognition and mood, and alterations in arousal and reactivity in response to exposure of a severe trauma to death, serious injury, or sexual activity (1). According to the Veteran Affairs, about 6% of the population will experience PTSD at any point in their lives.

Prazosin is a centrally acting alpha 1-adrenergic receptor antagonist often used off-label to counteract the hyperarousal and adrenergic dysregulation seen in PTSD. The medication has been shown to effectively reduce the effects of increased systemic norepinephrine release and norepinephrine receptor sensitivity seen in PTSD (2). By extension, the reduced effect of norepinephrine is theorized to also dampen the increased startle and fear responses and improve sleep quality. Improved sleep is especially significant as 70%–87% of patients with PTSD experience sleep disruption and 60%–90% experience insomnia (3, 4). Despite being documented as beneficial for many patients, there is some controversy regarding the effectiveness of prazosin. Raskind et al. and other advocates believe strongly that clinicians should consider prazosin, although one of their clinical trials showed no improvement in nightmares or sleep quality (5). In this trial, it was thought that selection bias of clinically stable patients less likely to benefit from prazosin possibly played a role, as well as the lack of screening for sleep apnea or sleep-disordered breathing which can mask beneficial effects of prazosin (5).

While the reduction of PTSD-related nightmares has been well-documented, there appears to be a return of nightmares upon prazosin discontinuation in trials (6). In a systematic review by Kung et al., it was noted that in several randomized controlled and open label studies, there were regular reports of distressing nightmares returning after discontinuation of prazosin (6); however, there are no reports to our knowledge documenting clear and consistent remission of nightmares after prazosin discontinuation. Unfortunately, the longest clinical trial for prazosin treatment of nightmares is 20 weeks, which may not be long enough of a treatment period for prazosin to have long-lasting effects (6).

In this case series, three patients with PTSD-related nightmares are treated successfully with prazosin and their nightmares do not return upon discontinuation of prazosin. We propose theories on the successful discontinuation of prazosin for these patients which may serve as a guide for clinicians planning to trial discontinuation of the medication due to side effects or intolerance.

## Case presentations

### Case 1

A female in her late 50’s with a past psychiatric history of major depressive disorder (MDD) and PTSD presented to the outpatient psychiatry clinic for treatment of depression. She endured daily crying spells, hopelessness, anger, hypervigilance, avoidance of trauma cues, flashbacks of prior trauma, nightmares, insomnia, and chronic passive suicidal ideation. She had a history of prior physical, sexual and verbal abuse. Her baseline Patient Health Questionnaire-9 (PHQ-9) was 18 out of 27 and she met criteria for MDD and PTSD. The patient had tried paroxetine for MDD/PTSD in the past, but it was discontinued due to jaw clenching. Her primary care physician started duloxetine 60 mg 4 months ago but she stopped taking it 2 months ago when she started noticing jaw clenching again. Her only psychiatric medication at intake was hydroxyzine 25 mg at bedtime as needed for insomnia. She had started weekly trauma-therapy 3 months prior and felt it was helpful. The psychiatrist recommended a retrial duloxetine at a low dose to target depression, PTSD, and chronic pain, but the patient declined. To target trauma-related flashbacks and nightmares, the psychiatrist prescribed prazosin 1 mg twice daily and continued hydroxyzine 25 mg at night as needed for insomnia. Her baseline blood pressure 2 days prior was 118/84 millimeters mercury (mmHg) and pulse was 78 beats per minute (bpm). Four blood pressures measurements were obtained over the prior month and blood pressure was stable per her primary care physician: the lowest was 110/78 mmHg and the highest was 134/97 mmHg.

Two weeks later, the patient presented to the clinic and reported only taking prazosin at night because she overslept the first morning and ended up deciding not to take the morning doses. Overall, the patient felt better and denied passive suicidal ideation. She denied nightmares since starting prazosin, but still reported flashbacks. She denied dizziness, lightheadedness, or any other side effects. She was agreeable to take prazosin 1 mg twice a day to treat flashbacks and agreed to start duloxetine 20 mg daily. Four months later, the patient denied flashbacks, nightmares, avoidance, or hypervigilance related to trauma. She denied side effects from prazosin and felt she no longer needed hydroxyzine for sleep. Blood pressure and pulse were measured as 111/77 mmHg and 89 bpm. Prazosin 1 mg twice daily and duloxetine 20 mg daily were continued. Blood pressure and pulse was measured monthly by the patient’s primary care physician over the following year: the lowest blood pressure over the following year was 107/75 mmHg and highest was 127/85 mmHg.

One year after starting prazosin 1 mg twice daily and duloxetine 20 mg daily, the patient requested to reduce her total number of medications and prazosin was decreased to 1 mg at bedtime. Her PHQ-9 at that time was 0. Blood pressure and pulse were measured as 127/82 mmHg and 77 bpm. Five months later, she denied recurrence of nightmares or flashbacks, was handling stressors in her life more effectively, and requested to stop prazosin completely. Prazosin was stopped, and duloxetine 20 mg daily and weekly trauma therapy were continued. Two years and 8 months after completely discontinuing prazosin, the patient continued to deny depression, flashbacks, nightmares, or other symptoms of PTSD. Her PHQ-9 score was maintained at 0.

### Case 2

A female in her early 70’s with a past psychiatric history of major depressive disorder and anxiety presented to the outpatient clinic for symptoms related to traumatic deaths of her son and husband over 10 years ago. She endured flashbacks and nightmares of prior trauma, hypervigilance, and avoidance of trauma cues. She denied symptoms of depression but at times felt life was not worth living when thinking about her trauma. Her baseline PHQ-9 was 5 out of 27 and she met criteria for MDD in partial remission and PTSD. In the past she took alprazolam 1 mg at bedtime for insomnia which reduced the frequency of her nightmares. Her psychiatric medications at intake included citalopram 60 mg daily for PTSD and quetiapine 25 mg at bedtime for insomnia. The psychiatrist continued her current medications and started prazosin 1 mg twice daily to target trauma-related flashbacks and nightmares. Her baseline blood pressure at this visit was 125/73 mmHg and pulse was 62 bpm. Blood pressure obtained a week prior was 128/72 mmHg and stable per her primary care physician. Trauma-focused therapy was also recommended, but the patient declined.

About 1 month after starting prazosin, the patient reported dizziness with blood pressure of 97/63 mmHg and pulse 62 bpm at her primary care physician’s office, so the psychiatrist discontinued the morning dose of prazosin and recommended a cardiology consultation. One month later, she reported a decrease in trauma-related nightmares with prazosin 1 mg at bedtime and denied dizziness. Her blood pressure was 126/64 mmHg and pulse was 61 bpm. She continued to report flashbacks and requested alprazolam for anxiety related to this. The psychiatrist increased prazosin to 2 mg at bedtime for nightmares and recommended monthly blood pressure monitoring. In addition, alprazolam 0.5 mg was prescribed daily as needed for anxiety. Citalopram 60 mg was changed to escitalopram 30 mg at bedtime to address concern of QTc prolongation per the cardiology consult.

Two months later, the patient reported that nightmares were “80% gone” with the increase in prazosin, and she denied dizziness or hypotension. She continued to report flashbacks but felt she could manage triggers without therapy. She continued to take alprazolam 0.5 mg daily as needed for anxiety about once per week after trauma cues and felt it was helpful. Her PHQ-9 at this visit was 1 out of 27 and her blood pressure was 120/67 mmHg. Prazosin was increased to 3 mg at bedtime for nightmares. Fourth months later, the patient denied nightmares and did not feel she needed quetiapine at night which was discontinued at this visit. Her blood pressure was 113/68 mmHg and continued to be measured monthly. About a year after starting prazosin 3 mg at bedtime, the patient reported increased flashbacks. She requested to try prazosin 1 mg daily in addition to 3 mg at bedtime (4 mg per day). Her blood pressure was 149/80 mmHg and pulse 88 bpm. She reported shortly afterwards that her flashbacks had stopped.

Four months after taking prazosin 1 mg daily and 3 mg at bedtime, the patient had a kidney biopsy after diagnosis of a kidney mass, and prazosin was discontinued after hemothorax and hypotension complications in the hospital. Prior to this hospitalization, her blood pressure was measured as 142/84 mmHg and it was unclear if prazosin contributed to the hypotension. She denied the return of nightmares or flashbacks after prazosin was discontinued. One and a half years after discontinuing prazosin, the patient continued to deny flashbacks, nightmares, or other symptoms of PTSD. Her PHQ-9 score was 0.

### Case 3

A female in her late 50’s with a past psychiatric history of major depressive disorder and generalized anxiety disorder presented to the outpatient psychiatry clinic for poor sleep and symptoms related to past trauma. She reported nightmares with fear of going to sleep, flashbacks, and anxiety related to prior physical abuse. Her baseline PHQ-9 was recorded as 14 out of 27, 1 month prior by her therapist; however, recorded symptoms were due to nightmares and flashbacks from PTSD. She did meet criteria for PTSD and MDD in full remission. Her psychiatric medications at intake included fluoxetine 20 mg daily for mood, aripiprazole 20 mg daily for mood, and trazodone 50 mg at bedtime as needed for insomnia. She reported the aripiprazole may have caused an occasional tremor but was unsure. The psychiatrist increased her fluoxetine to 30 mg daily for PTSD symptoms and started prazosin 1 mg at bedtime to target trauma-related nightmares. Aripiprazole was continued at 20 mg daily. Her most recent blood pressure was 162/70 mmHg and pulse 68 bpm. Her blood pressure the month prior was 151/74 mmHg and stable per her primary care physician. She was already in weekly trauma-focused therapy and found it helpful. One month after starting prazosin she denied nightmares or flashbacks and reported her sleep had improved. Her blood pressure was 145/85 mmHg and continued to be measured monthly by her primary care physician. She requested the medications be kept the same due to grieving a death in the family at that time.

Eight months after starting prazosin, the patient continued to deny trauma-related nightmares and flashbacks, and only complained about occasional night-time awakenings. Shortly after this appointment, a family member suffered a medical emergency, and she reported new nightmares related to this. Her blood pressure was 162/88 mmHg. Prazosin was increased to 2 mg at bedtime, and 2 months later she denied nightmares, reported sleeping 8 h each night, and her PHQ-9 score was 0. Her blood pressure was measured as 155/86 mmHg. At this visit, the patient complained of worsening hand tremor. She was started on primidone 100 mg twice daily by her neurologist and aripiprazole was slowly tapered to 2 mg daily over the next 2 years due to possible contribution to the tremor. Blood pressure was measured monthly over the next 2 years: the lowest blood pressure was 110/90 mmHg and highest was 162/88 mmHg.

More than 2 years after taking prazosin 2 mg at bedtime, the patient reported flashbacks and nightmares were returning after her boyfriend threatened her. Her blood pressure was 134/78 mmHg. Prazosin was increased to 1 mg twice daily (morning and afternoon) for flashbacks and 2 mg at bedtime for nightmares. One month later she denied flashbacks or nightmares and her blood pressure was measured as 134/72 mmHg. Unexpectedly, all of the patient’s psychotropics, including fluoxetine, aripiprazole, prazosin, and trazodone were discontinued and not restarted during hospitalization for somnolence due to possible contribution to the condition. Four months later, she reported some anxiety but denied nightmares or flashbacks. She requested fluoxetine be restarted for anxiety. The psychiatrist prescribed fluoxetine 20 mg daily and trazodone 50 mg at bedtime as needed for insomnia. Ten months after prazosin was discontinued, the patient continued to deny flashbacks, nightmares, or other symptoms related to prior trauma. Her PHQ-9 score was 0. She felt her mood was good while taking fluoxetine and participating in trauma-focused therapy.

## Discussion

The primary treatment for PTSD is trauma-informed cognitive behavioral therapy (7). For patients that prefer pharmacotherapy or do not have access to therapy, the medications approved by the Food and Drug Administration for PTSD are the selective serotonin reuptake inhibitors (SSRIs) sertraline and paroxetine (8). The Veteran Affairs also recommends the SSRI paroxetine and the serotonin norepinephrine reuptake inhibitor (SNRI) venlafaxine (7, 9). Off-label medications for PTSD include anti-adrenergic agents such as prazosin, atypical antipsychotics, and mood stabilizers (10).

Prazosin is an alpha-adrenergic antagonist often prescribed off-label for PTSD-related nightmares. It is worth noting that there is variation in recommendations for how to utilize prazosin for PTSD. In a systematic review of PTSD treatments by Martin et al., prazosin was noted to be first-line recommended treatment for PTSD-related nightmares in two guidelines, while other guidelines recommended it as third-line or provided no guidance at all (11). In addition, the effective dose of prazosin for nightmares tends to vary in case reports from 0.25 mg up to 50 mg daily (12, 13). Recommendations based on guidelines suggest that prazosin be started at a low dose and titrated to an effective range of 3–16 mg/night (14); however, long-term efficacy has not been established. In our case series, one patient’s final daily dose of prazosin was 1 mg and the two other patients’ final daily doses were 4 mg, divided into two or three doses. Each patient reported a complete resolution in PTSD symptoms at these doses, suggesting that some patients may not require higher doses for prazosin to be effective.

First-dose hypotension is a known phenomenon of prazosin and other alpha-antagonists that leads to postural hypotension and possible syncope after the first dose of the medication. It is more common in patients taking beta blockers or diuretics. This side effect of prazosin has been minimized in PTSD clinical trials by utilizing a small starting dose of 1 mg and slowly titrating by 1 mg every few days (15). None of the patients in our case series experienced first-dose hypotension; however, one patient did experience hypotension and dizziness within a few weeks. In all patients in this case series, there were standing medications that increased risk of hypotension when prazosin was added. In the first case, duloxetine, an SNRI, was added which has additive effects with prazosin and may increase risk of hypotension; fortunately, the patient did not experience any side effects, but blood pressure was monitored monthly. This patient was also taking hydroxyzine 25 mg, but there are no known interactions with prazosin. In the second case, the patient was taking citalopram (later changed to escitalopram due to concern for QTc prolongation) and quetiapine when prazosin was added twice daily. Quetiapine and prazosin have additive effects and can increase the risk of hypotension including orthostasis and syncope. This patient did experience symptomatic hypotension, likely due to this increased risk, and the prazosin dose was decreased. Prazosin was then able to be more slowly titrated and quetiapine was eventually discontinued. In the scenario of a co-occurring medication with risk of hypotension, it would be better to start prazosin at once daily instead of twice daily and with slower titration. Alprazolam was also added at bedtime which has no known interactions with prazosin. In the third case, the patient was taking fluoxetine, aripiprazole, and trazodone when prazosin 1 mg at bedtime was added. Like quetiapine, aripiprazole also has the increased risk of hypotension when added with prazosin. However, the patient did not have any side effects from the addition of prazosin, likely due to starting at 1 mg and a slower titration. In addition, the dose of aripiprazole was lowered to 2 mg due to tremor as prazosin was titrated, likely contributing to better tolerance of the medication.

In each of these cases, there were multiple similarities which could account for the resolution of flashbacks and nightmares related to PTSD; however, there were also many differences further implicating the import role of prazosin (Table 1). In the first and third case, trauma-based therapy was already in motion before prazosin was even started, which could lead to the resolution of symptoms itself (16). However, the patient in the second case declined therapy and still had resolution of flashbacks and nightmares. The number of years the patients reported experiencing PTSD symptoms prior to starting prazosin varied greatly and did not appear to impact treatment (Table 1). The main similarity between all three cases are prazosin combined with an SSRI or SNRI. In the second and third case, the patients’ PTSD symptoms had not resolved fully even after having been prescribed an SSRI prior to the first appointment, suggesting that prazosin has a role to play in reducing these symptoms.

**Table 1:** Comparison of timelines between the three cases.

	Case 1	Case 2	Case 3
Approximate # of years of PTSD symptoms prior to prazosin	51	20	6
SSRI/SNRI prior to prazosin	None	Citalopram	Fluoxetine
Trauma-focused therapy	Yes	No	Yes
Starting prazosin dose	1 mg AM and HS	1 mg AM and HS	1 mg HS
Last prazosin dose	1 mg at HS	1 mg AM and 3 mg HS	1 mg AM, 1 mg in afternoon, and 2 mg HS
Approximate # of months until prazosin discontinued	21	20	42
Approximate # of months of remission after prazosin discontinued	32	18	10

AM: in the morning; HS: at night.

All three patients described nightmares and flashbacks as disturbing symptoms which prazosin has been shown to target. We propose that the increase in serotonin/norepinephrine in the brain responsible for long-term brain changes along with decreased autonomic arousal from prazosin led to resolution of PTSD symptoms. SSRIs increase serotonin in the brain and SNRIs additionally increase norepinephrine, but the exact treatment mechanisms for PTSD are unknown. In a study by Fernandez et al., SSRI treatment increased activation in the dorsolateral prefrontal cortex and supplementary motor area during emotion regulation after 6 months of treatment (17). In comparison, symptom reduction with prazosin occurs within a few weeks by preventing the norepinephrine-induced release of corticotropin releasing factor and reducing flight or fight and startle response (18).

In clinical trials by Raskind et al., the discontinuation of prazosin led to the recurrence of PTSD symptoms; however, the average length of these trials is 8 weeks (4, 5, 19, 20). The understanding of varying prazosin doses on the discontinuation and remission of PTSD are limited currently. The patients in the above cases each achieved continued remission of PTSD symptoms despite varied dosing that ranged between 1 mg nightly to 4 mg daily split between two or three doses, suggesting that a high dose is not needed for remission upon discontinuation. The most significant aspect of the cases in this series are the combination of an SSRI or SNRI with prazosin within an average of 1 to 3 years, which allowed the patients to discontinue prazosin without recurrence of PTSD symptoms. In one study by Ketenci et al., treatment with prazosin increased norepinephrine levels significantly in the amygdala and rostral pons and thus increased GABA, thought to inhibit fear conditioning in stressed rats (21). This was thought to decrease stress and defensive behaviors before a trauma stimulus (21). The clinical trial timeline of 8 weeks is likely not enough time for long-term changes in the brain from prazosin to come to effect. These three cases support that the synergistic effect of SSRI/SNRI with prazosin for about 2 years (average 28 months) prior to discontinuation of prazosin might be enough time for permanent brain changes to persist (Table 1).

A limitation of this case series is that it is a small sample size of patients seen by only one psychiatrist. However, the patients in this case provide unique cases that have not been published in the past regarding successful remission of PTSD symptoms after discontinuation of prazosin. This case series serves to add to the literature of prazosin utilized for treatment of PTSD symptoms and can be used as a guide for discontinuation of the medication. Continued research on the effects of prazosin discontinuation and remission of PTSD is recommended.

## Conclusion

This case series involved the treatment of PTSD with recommended psychotherapy and an SSRI or SNRI along with addition of prazosin for distressing arousal symptoms including flashbacks and nightmares. Combining an SSRI or SNRI with prazosin for an average of about 28 months before discontinuation of prazosin may prevent recurrence of symptoms. This case serves to provide guidance as to when a trial of prazosin discontinuation may be warranted due to intolerance, side effects, or reducing polypharmacy. Further studies would be helpful in elucidating these trends to promote further guidance in how to prevent re-emergence of flashbacks and nightmares due to PTSD after successful treatment.

## Patient perspective

Case 2: “I felt fine. I did not have any side effects (from prazosin) … there were no negative effects … it worked over a period of time. It alleviated the nightmares and helped me sleep. Before, I would go to sleep but I would wake with horrific nightmares and would shake from head to toe, and then I would not be able to go to sleep. With prazosin, I wasn’t having nightmares anymore for quite a while. I wanted to see if I could go without the prazosin and if they would stay away, and they did. There was no breakthrough. The prazosin took care of nightmares long-term.”

## Data availability statement

The original contributions presented in the study are included in the article/supplementary material, further inquiries can be directed to the corresponding author.

## Ethics statement

Written informed consent was obtained from the individual(s) for the publication of any potentially identifiable images or data included in this article.

## Author contributions

CR, JY, JS, and MF contributed equally to the formulation of these cases. All authors contributed to the article and approved the submitted version.

## Funding

APC was funded by Rowan University Open Acccess Publishing Fund.

## Conflict of interest

The authors declare that the research was conducted in the absence of any commercial or financial relationships that could be construed as a potential conflict of interest.

## Publisher’s note

All claims expressed in this article are solely those of the authors and do not necessarily represent those of their affiliated organizations, or those of the publisher, the editors and the reviewers. Any product that may be evaluated in this article, or claim that may be made by its manufacturer, is not guaranteed or endorsed by the publisher.
